# Efficient production of bacterial antibiotics aminoriboflavin and roseoflavin in eukaryotic microorganisms, yeasts

**DOI:** 10.1186/s12934-023-02129-8

**Published:** 2023-07-20

**Authors:** Kostyantyn V. Dmytruk, Justyna Ruchala, Liubov R. Fayura, Grzegorz Chrzanowski, Olena V. Dmytruk, Andriy O. Tsyrulnyk, Yuliia A. Andreieva, Daria V. Fedorovych, Olena I. Motyka, Diethard Mattanovich, Hans Marx, Andriy A. Sibirny

**Affiliations:** 1 Institute of Cell Biology, National Academy of Sciences of Ukraine, Drahomanov St, 14/16, Lviv, 79005 Ukraine; 2 University of Rzeszow, Zelwerowicza 4, Rzeszow, 35-601 Poland; 3 Research Institute of Epidemiology and Hygiene of the Danylo Halytsky Lviv National Medical University, Zelena St, 12, Lviv, 79005 Ukraine; 4 Department of Biotechnology, Institute of Microbiology and Microbial Biotechnology, University of Natural Resources and Life Sciences, Muthgasse 18, Vienna, Vienna, 1190 Austria

**Keywords:** Yeast, Antibiotics, Metabolic engineering

## Abstract

**Background:**

Actinomycetes *Streptomyces davaonensis* and *Streptomyces cinnabarinus* synthesize a promising broad-spectrum antibiotic roseoflavin, with its synthesis starting from flavin mononucleotide and proceeding through an immediate precursor, aminoriboflavin, that also has antibiotic properties. Roseoflavin accumulation by the natural producers is rather low, whereas aminoriboflavin accumulation is negligible. Yeasts have many advantages as biotechnological producers relative to bacteria, however, no recombinant producers of bacterial antibiotics in yeasts are known.

**Results:**

Roseoflavin biosynthesis genes have been expressed in riboflavin- or FMN-overproducing yeast strains of *Candida famata* and *Komagataella phaffii*. Both these strains accumulated aminoriboflavin, whereas only the latter produced roseoflavin. Aminoriboflavin isolated from the culture liquid of *C. famata* strain inhibited the growth of *Staphylococcus aureus* (including MRSA) and *Listeria monocytogenes*. Maximal accumulation of aminoriboflavin in shake-flasks reached 1.5 mg L^− 1^ (*C. famata*), and that of roseoflavin was 5 mg L^− 1^ (*K. phaffii*). Accumulation of aminoriboflavin and roseoflavin by *K. phaffii* recombinant strain in a bioreactor reached 22 and 130 mg L^− 1^, respectively. For comparison, recombinant strains of the native bacterial producer *S. davaonensis* accumulated near one-order less of roseoflavin while no recombinant producers of aminoriboflavin was reported at all.

**Conclusions:**

Yeast recombinant producers of bacterial antibiotics aminoriboflavin and roseoflavin were constructed and evaluated.

**Supplementary Information:**

The online version contains supplementary material available at 10.1186/s12934-023-02129-8.

## Introduction

**Fig. 1:**
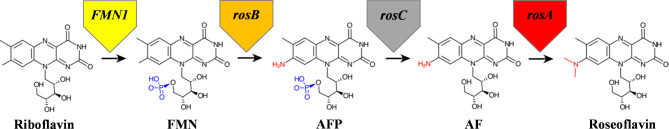
Roseoflavin biosynthesis pathway of *S*. *davaonensis* expressed in yeasts [5]. Genes *FMN1, rosB, rosC* and *rosA* encoding riboflavin kinase (EC 2.7.1.26), 8-demethyl-8-amino-riboflavin-5′-phosphate synthase (EC 2.6.1.114), AF-phosphate phosphatase and dimethyltransferase (EC 2.1.1.343), respectively, and also are responsible for synthesis of roseoflavin. FMN (flavin mononucleotide), AFP (8‑demethyl‑8‑amino‑riboflavin‑5′‑phosphate) and AF (8‑demethyl‑8‑amino‑riboflavin)

Roseoflavin (RoF, 7-methyl-8-dimethylamino-(1**’**-D-ribityl)isoalloxazine) is a natural antibiotic flavin of reddish-orange color, which was isolated in *Streptomyces davaonensis* and *Streptomyces cinnabarinus* [1, 2]. Biochemical pathways of RoF biosynthesis were deciphered by the group of M. Mack at the University of Mannheim, Germany (Fig. 1) [3]. The group identified gene *rosB*, which encodes the enzyme converting flavin mononucleotide (FMN) to 8-demethyl-8-aminoriboflavin-5’-phosphate (short name, aminoriboflavin-5’-phosphate, AFP; [3]). AFP is dephosphorylated to aminoriboflavin (AF) by a specific phosphatase, the product of *S. davaonensis* gene *rosC* [4]. Finally, AF is dimethylated to RoF using an enzyme encoded by *rosA* [2].

The effects of RoF and its immediate biosynthetic precursor AF on the bacterial growth were studied. RoF effectively inhibits growth of many Gram-positive bacteria, e.g. *Staphylococcus aureus* (including methicillin-resistant *S. aureus*, MRSA), *Listeria monocytogenes, Clostridium difficile*, *Enterococcus faecalis, Bacillus subtilis*, some Gram-negative bacteria capable of riboflavin transport and others [1, 6–10]. To inhibit growth of MRSA, a 16-fold lower RoF concentration is needed than for linezolid [11, 12]. RoF is also promising for use as an effective medicine against parasitic protozoa, including malaria plasmodia, coccidia, and trypanosomes [13, 14]. RoF inhibition is caused by its conversion to the analogs of flavin nucleotides RoFMN and RoFAD, which cannot act as coenzymes. RoFMN in bacteria also acts as a riboswitch effector that blocks riboflavin biosynthesis *de novo* and riboflavin transport [7, 15, 16]. Unfortunately, RoF proved toxic to human cells due to its conversion to analogs of flavin coenzymes. At the same time, immediate biosynthetic precursor of RoF, AF inhibit Gram-positive bacteria, whereas it showed little or no toxicity to human cells [6, 7, 17], which could be explained by its poor conversion to analogs of flavin nucleotides in mammal cells. It should be noted that derivatives of RoF show anticancer activity [18]. Thus, both flavin antibiotics, RoF and AF, seem promising candidates for the development of effective antibacterial or anticancer drugs. This should be achieved by construction of strains with substantially elevated production of these antibiotics, which could then be chemically modified for improvement to give them specificity and efficacy. The natural bacterial producer *S. davaonensis* synthesizes up to 8 mg L^-1^ RoF [5] and negligible amounts of AF. Metabolic engineering increased RoF synthesis in the natural producer, *S. davaonensis*, by up to 14 mg L^-1^ after 10 days of cultivation [5]. RoF biosynthesis genes have not been functionally expressed in the riboflavin overproducing strains of *Bacillus subtilis* for unknown reasons. At the same time, overexpression of these genes was successful in *Corynebacterium glutamicum*, and resulted in RoF production in small amounts, of up to 0.7 mg L^-1^ [5]. No strains overproducing AF have been reported.

Yeasts have substantial advantages over bacteria for biotechnological use as they usually have simple nutrition requirements, fast growth and resistance to viral infections. Yeasts are easier to separate from cultural liquid as their cells are larger and heavier compared to bacteria. Contrary, actinobacteria, including representatives of genus *Streptomyces*, are characterized by poor growth and susceptibility to phage contamination and lysis. However, none of yeast producers of bacterial antibiotics have been constructed so far. Yeast organisms have only been explored to produce the fungal antibiotic penicillin, naturally produced by *Penicillium chrysogenum.* Such recombinant yeast strains accumulated much less penicillin relative to the wild-type of *P. chrysogenum* [19, 20]. At the same time, no yeast producers of prokaryotic antibiotics are known.

We constructed producers of flavin antibiotics AF and RoF based on riboflavin and flavin mononucleotide overproducing strains previously isolated in our laboratories. One organism was the yeast *Candida famata* (also called, anamorph, *Candida flareri*, teleomorph, *Debaryomyces subglobosus*; [21, 22]). *C. famata* is a representative of so-called flavinogenic yeasts that overproduce riboflavin under conditions of iron starvation and is, apparently, the most active riboflavin producer among this group of yeast species [23]. Riboflavin overproducing strains of *C. famata* are known [24–28], which have been used for industrial production of this vitamin. Moreover, we constructed unique *C. famata* strains overproducing FMN [29], which are promising parental strains for constructing AF and RoF producers. The second host yeast used in this work was *Komagataella phaffii* (*Pichia pastoris*), which belongs to the best studied yeasts being the favorite model system for cell biology [30, 31], production of recombinant proteins [32, 33], and secondary metabolites such as lovastatin, β-carotene and astaxanthin [34]. Previously the yeast *K. phaffii* had been engineered to overproduce riboflavin [35], so the corresponding strains could be attractive hosts for flavin antibiotic synthesis. Herein we report on the successful use of riboflavin and FMN overproducing strains of *C. famata* and riboflavin overproducing strains of *K. phaffii* for constructing AF and RoF producing yeast strains. These accumulated the highest titers AF and RoF described so far.

## Results

### Expression of the AF/RoF biosynthetic genes in *C. famata*

Since the flavin antibiotic synthesis starts from riboflavin, it was tempting to use flavinogenic yeast as a parental organism for construction of efficient producers of these compounds. The promising organism of choice is the flavinogenic yeast *C. famata*, which is capable of riboflavin oversynthesis [23]. Recombinant strains of *C. famata* overproducing riboflavin and FMN constructed in our lab were used as parental strains. Codon optimized gene *rosB* from *S. davaonensis* encoding an 8-demethyl-8-aminoriboflavin-5′-phosphate (AFP) synthase under the control of strong constitutive *TEF1* promoter was integrated into the genome of FMN overproducing strain FP (for description of all the strains see Supplementary Table 1). Overexpression of *rosB* in strain FP/rosB was confirmed by qPCR (Supplementary Table 2).

To determine AF production by *C. famata* FB/rosB, this strain was grown in YNB medium with a starting OD_600_ of 5 for 72 h. FB/rosB accumulated riboflavin, FMN and the novel flavin (Table 1). This flavin was purified in 2-step column chromatography, first with Florisil and second with cellulose. Purified flavin was analyzed by mass spectrometry.

**Table 1:** Biomass (CDW), riboflavin, FMN, aminoriboflavin, roseoflavin production and dimethyltransferase specific activity of *C. famata* and *K. phaffii* transformants and control strains represented on YNB medium on 72 h of cultivation

Strain	CDW (g L^-1^)	Riboflavin (mg L^-1^)	FMN (mg L^-1^)	AF (mg L^-1^)	RoF (mg L^-1^)	RosA specific activity (U mg^-1^)
FP	2.8 ± 0.1	301.1 ± 15.2	5.1 ± 0.2	ND	ND	NA
FP/rosB	2.7 ± 0.1	328.4 ± 15.4	3.2 ± 0.1	1.5 ± 0.1	ND	NA
BRP	2.7 ± 0.1	789.3 ± 38.2	ND	ND	ND	ND
BRP/FMN1-rosB-rosA-rosC	2.6 ± 0.1	841.3 ± 40.4	1.1 ± 0.05	1.5 ± 0.1	ND	ND
Y-33	2.1 ± 0.1	2.3 ± 0.1	ND	ND	ND	ND
Y-33/FMN1-rosB	2.2 ± 0.1	4.1 ± 0.2	0.1	1.9 ± 0.1	ND	NA
Y-33/FMN1-rosB-rosA-rosC	2.1 ± 0.1	7.8 ± 0.4	0.1	2.0 ± 0.1	5.0 ± 0.2	24.1 ± 1.2
Y-33 (bioreactor 165 h)*	142.9 ± 7.0	388.0 ± 8.7	ND	ND	ND	NA
Y-33/FMN1-rosB (bioreactor 96 h)*	130.0 ± 6.4	564.0 ± 27.9	0.9 ± 0.04	17.0 ± 0.8	ND	NA
Y-33/FMN1-rosB-rosA-rosC (bioreactor 312 h)*	133.0 ± 6.6	1625.4 ± 82.1	1.0 ± 0.05	21.8 ± 1.0	129.6 ± 6.3	NA

ND, not detected;

NA, not applicable;

*Conditions of high cell density cultivation in bioreactor, described in Section “Fed-batch fermentations”

**Fig. 2:**
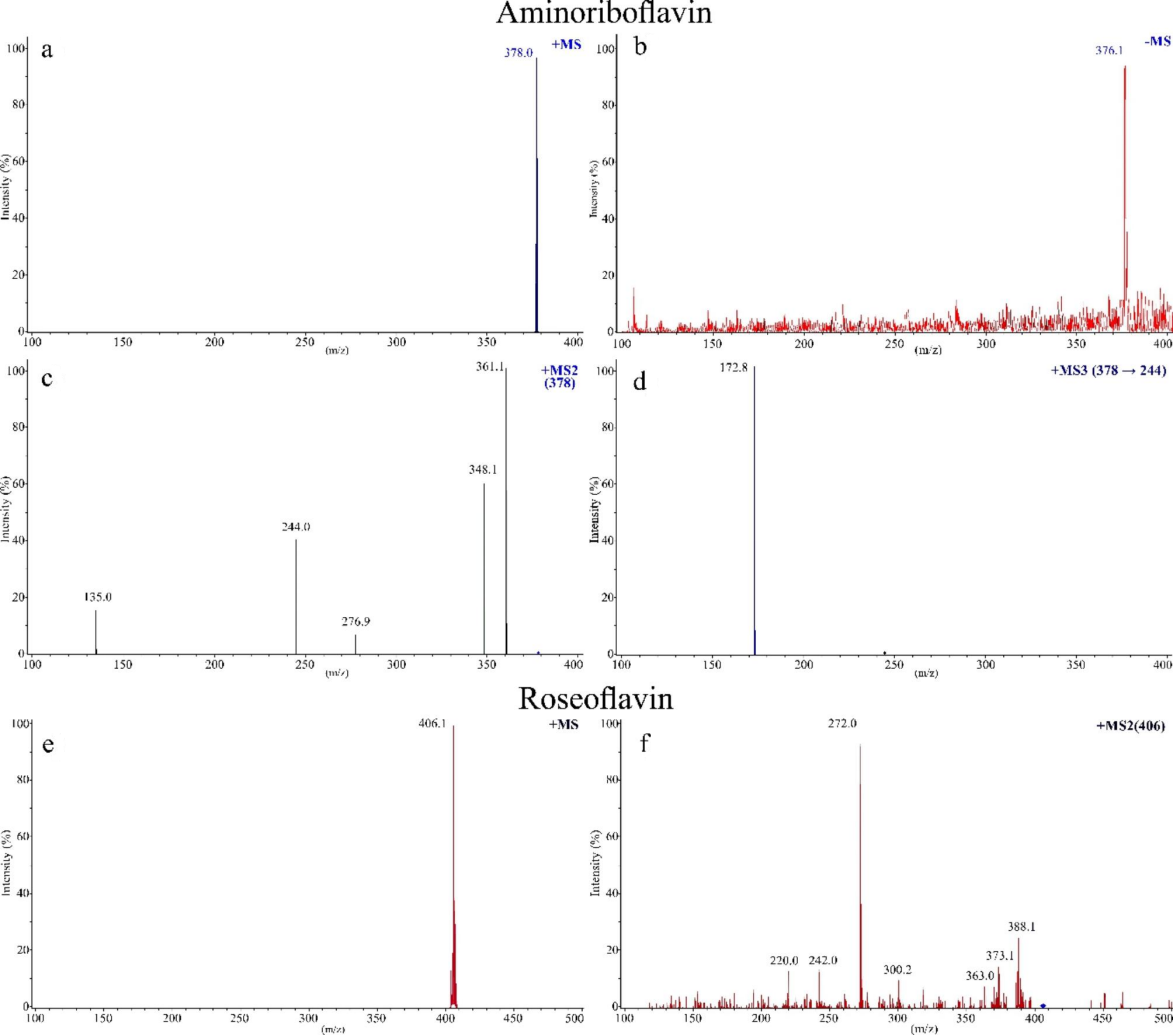
Mass spectrometry analysis of aminoriboflavin and roseoflavin produced by yeast recombinant strains; **(a)** Molecular ion of aminoriboflavin [M + H] in positive ionization mode, **(b)** Molecular ion of aminoriboflavin [M-H] in negative ionization mode; **(c)** MS/MS fragmentation of aminoriboflavin in positive ionization mode; **(d)** Fragmentation of ion at m/z 244 (aminolumichrome); **(e)** Molecular ion of roseoflavin [M + H] in positive ionization mode, **(f)** MS/MS fragmentation of roseoflavin in positive ionization mode

The ESI-MS spectrum of the novel purified flavin with elemental composition C_16_H_19_N_5_O_6_ (Fig. 2a) showed an intense protonated molecular ion [M + H] at m/z 378 (elemental composition C_16_H_20_N_5_O_6_). In negative ionization mode, a molecular ion [M - H] at m/z 376 with an elemental composition C_16_H_18_N_5_O_6_ was produced (Fig. 2b). Aminolumichrome with a molecular mass at m/z 244 was detected after MS/MS fragmentation of AF (Fig. 2c) instead of 243 m/z for lumichrome that was found after fragmentation of riboflavin [36]. The data suggest that the amino group replaced the methyl group of riboflavin. Moreover, fragmentation of the parent ion the presence of an ion at 135 m/z related to a 4-hydroxylated 5-carbon chain. Furthermore, fragmentation of aminolumichrome (244 m/z) from AF had an ion of mass 172.8 (Fig. 2d).

One predominant compound at the retention time of 3.373 min was found on the base of chromatographic analysis using HPLC-DAD system (Supplementary Fig. 1a). Moreover, the UV-VIS spectrum recorded for that peak (Supplementary Fig. 1b) had a shape with a maximum at 476 nm. The same maximum has been found by [37] for AF without RosA protein and the further decrease in the absorbance after the addition of RosA protein. Obtained results clearly showed that purified flavin is AF.

**Fig. 3:**
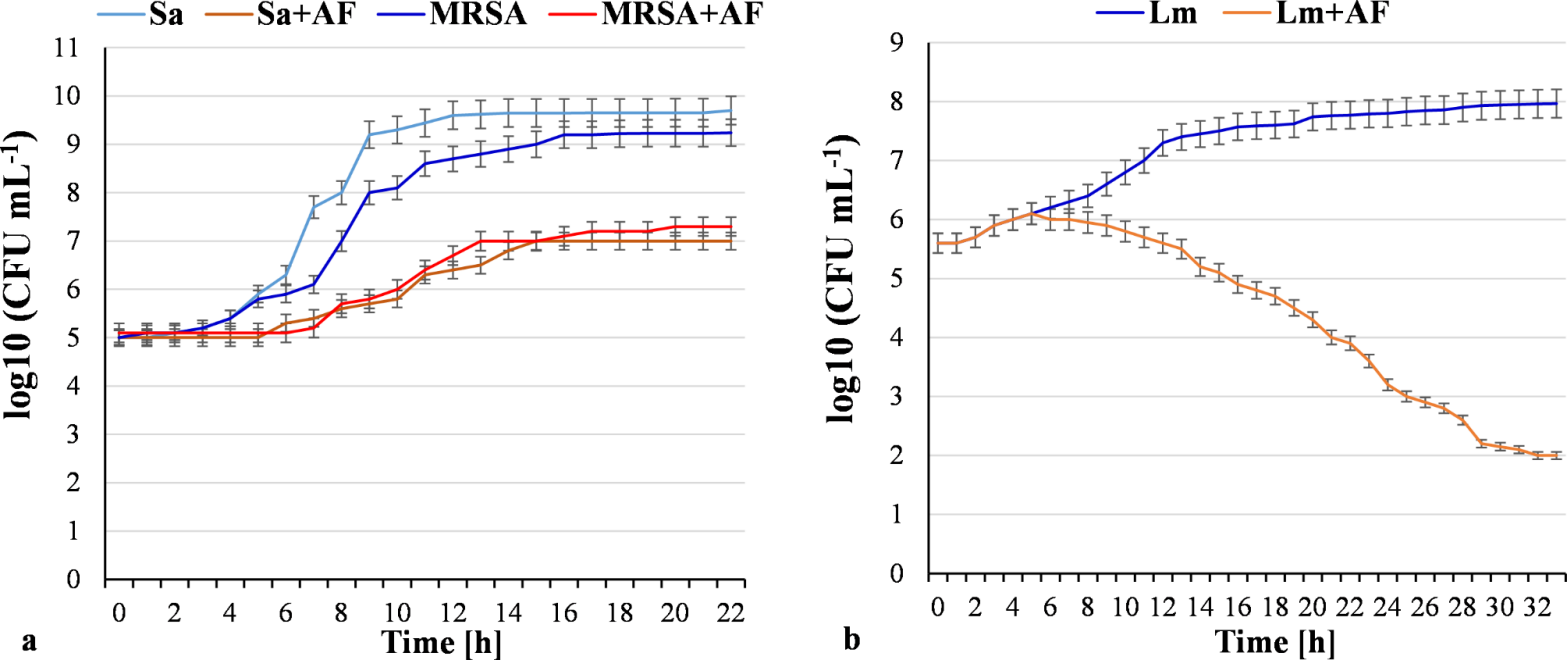
Growth inhibition of *Staphylococcus aureus* (Sa) ATCC 25,923 and MRSA ATCC 43,300 **(a)** and *Listeria monocytogenes* (Lm) ATCC 19,113 **(b)** by 200 mg L^-1^ of aminoriboflavin (AF)

Quantitative assay showed that FP/rosB accumulated 1.5 mg of AF L^-1^. FP/rosB produced 3.2 mg of FMN L^-1^, which is 1.6-fold less compared to the parental strain (Table 1). The decrease in FMN production can be explained by AF accumulation. Preparative quantities of the AF from yeast culture were accumulated and purified. AF was used to test the growth inhibition of *S. aureus* and *L. monocytogenes.* 200 mg L^-1^ of AF showed similar bacteriostatic activity against *S. aureus* ATCC 25,923 and MRSA ATCC 43,300 (Fig. 3a). 200 mg L^-1^ AF also had strong bactericidal activity against *L. monocytogenes* ATCC 19,113 (Fig. 3b).

To construct the RoF producer gene *FMN1* from closely related species, *D. hansenii* coding for riboflavin kinase and RoF biosynthetic genes from *S. davaonensis*, namely *rosB, rosC* (AF-phosphate phosphatase) and *rosA* (dimethyltransferase) under the control of *TEF1* promoter were integrated into the genome of the riboflavin overproducing strain BRP. Expression of *FMN1*, *rosB*, *rosC* and *rosA* genes in strain BRP/FMN1-rosB-rosA-rosC was confirmed by qPCR (Supplementary Table 2). Expression of *FMN1* was similar to that of FB/rosB. Expression of *rosB* in BRP/FMN1-rosB-rosA- rosC was 2.7-fold higher compared to the FB/rosB strain. Expression of *rosC* was comparable to that of *rosB*, whereas expression of *rosA* was ~ 2-fold higher than those of *rosB* or *rosC* (Supplementary Table 2).

To estimate flavin production, the constructed strain BRP/FMN1-rosB-rosA-rosC was cultured in shake flask system. Despite proper expression of the RoF biosynthetic genes, no RoF production was found. However, BRP/FMN1-rosB-rosA-rosC produced 1.5 mg AF L^-1^ similar to AF production by FP/rosB strain (Table 1). BRP/FMN1-rosB-rosA-rosC accumulated 1.1 mg L^-1^ of FMN confirming expression of *FMN1* gene. To answer the question as to why BRP/FMN1-rosB-rosA-rosC does not produce RoF, we assayed specific dimethyltransferase (RosA) activity, but did not find any activity (Table 1). Possible explanations for the lack of dimethyltransferase activity in the strain could be improper folding of the RosA or the presence of some specific inhibitors of this enzyme in *C. famata*.

### Expression of the AF/RoF biosynthetic genes in *K. phaffii*

*K. phaffii* is widely used in industry as a host system for heterologous protein expression. As an alternative host for the construction of RoF producer, we used the riboflavin overproducing strain Y-33 of *K. phaffii*, which was constructed by overexpression of all structural genes involved in synthesis of vitamin B2.

For construction of the AF producer, 2 expression cassettes containing the homologous gene *FMN1* under the control of *TEF1* promotor and optimized *rosB* gene under control of the *GAP1* promotor were integrated into the genome of Y-33. The constructed Y-33/FMN1-rosB strain produced 1.9 mg AF L^-1^ (Table 1). Y-33/FMN1-rosB increased by 1.8-fold in riboflavin production compared to Y-33, amounting to 4.1 mg L^-1^ (Table 1).

The RoF producing strain of *K. phaffii* was constructed by multicopy overexpression of genes *FMN1* from *K. phaffii*, and *rosB*, *rosC* and *rosА* from *S. davaonensis.* The principal of multicopy overexpression is described by [38]. RoF biosynthetic genes were placed under the control of the *GAP1* promotor. Multicopy integration was achieved by using NTS locus in frame of the corresponding integrative plasmids bearing a selective marker conferring resistance to antibiotic nourseothricin (NTC). Strain Y-33/FMN1-rosB-rosA-rosC was selected by transferring it to agar plates with gradually increasing the NTC concentration. The overexpression of target genes was confirmed by qPCR. Expression of *FMN1* was 4.2-fold higher than that in Y-33/FMN1-rosB (Supplementary Table 2). Expression of *rosB* increased 3.9-fold compared strain Y-33/FMN1-rosB. Expression of *rosC* and of *rosA* were similar, exceeding the expression of *rosB* by 1.6-fold (Supplementary Table 2). The specific activity of the dimethyltransferase in Y-33/FMN1-rosB-rosA-rosC was determined. RosA activity was 24.1 units / mg protein, whereas the engineered strain of *C. famata* BRP/FMN1-rosB-rosA-rosC had no activity (Table 1). The data confirmed our assumption that *K. phaffii* is a better host than *C. famata* host for heterologous protein expression.

During flask cultivation, strains Y-33/FMN1-rosB-rosA-rosC produced slightly higher amount of AF than Y-33/FMN1-rosB reaching 2 mg L^-1^, presumably, as a result of a 3.9-fold higher expression of *rosB* (Supplementary Table 2). Both strains produced minor amount of FMN. The strain Y-33/FMN1-rosB-rosA-rosC had a 3.4-fold or 1.9-fold increase in riboflavin production when compared to that of the strains Y-33 and Y-33/FMN1-rosB, respectively (Table 1). Remarkably, strain Y-33/FMN1-rosB-rosA-rosC was able to produce 5 mg L^-1^ of RoF (Table 1).

The ESI-MS spectrum of RoF (Fig. 2e) showed intense protonated molecular ions [M + H] at m/z 406.1 (elemental composition C_18_H_23_N_5_O_6_). Fragmentation of this molecular ion showed (Fig. 2f) an intensive daughter ion at 272 m/z (derived after elimination of its 5-carbon chain). Moreover, less weak signals were found for daughter ions at m/z 388 (water elimination), 373 (dismissal of water and methyl group) and 363 (elimination of dimethylamine group at position 8). Chromatogram and UV-VIS spectrum of RoF produced by Y-33/FMN1-rosB-rosA-rosC perfectly overlapped with that of RoF standard (Sigma-Aldrich) (Supplementary Fig. 2a, 2b).

### High cell density cultivations

**Fig. 4:**
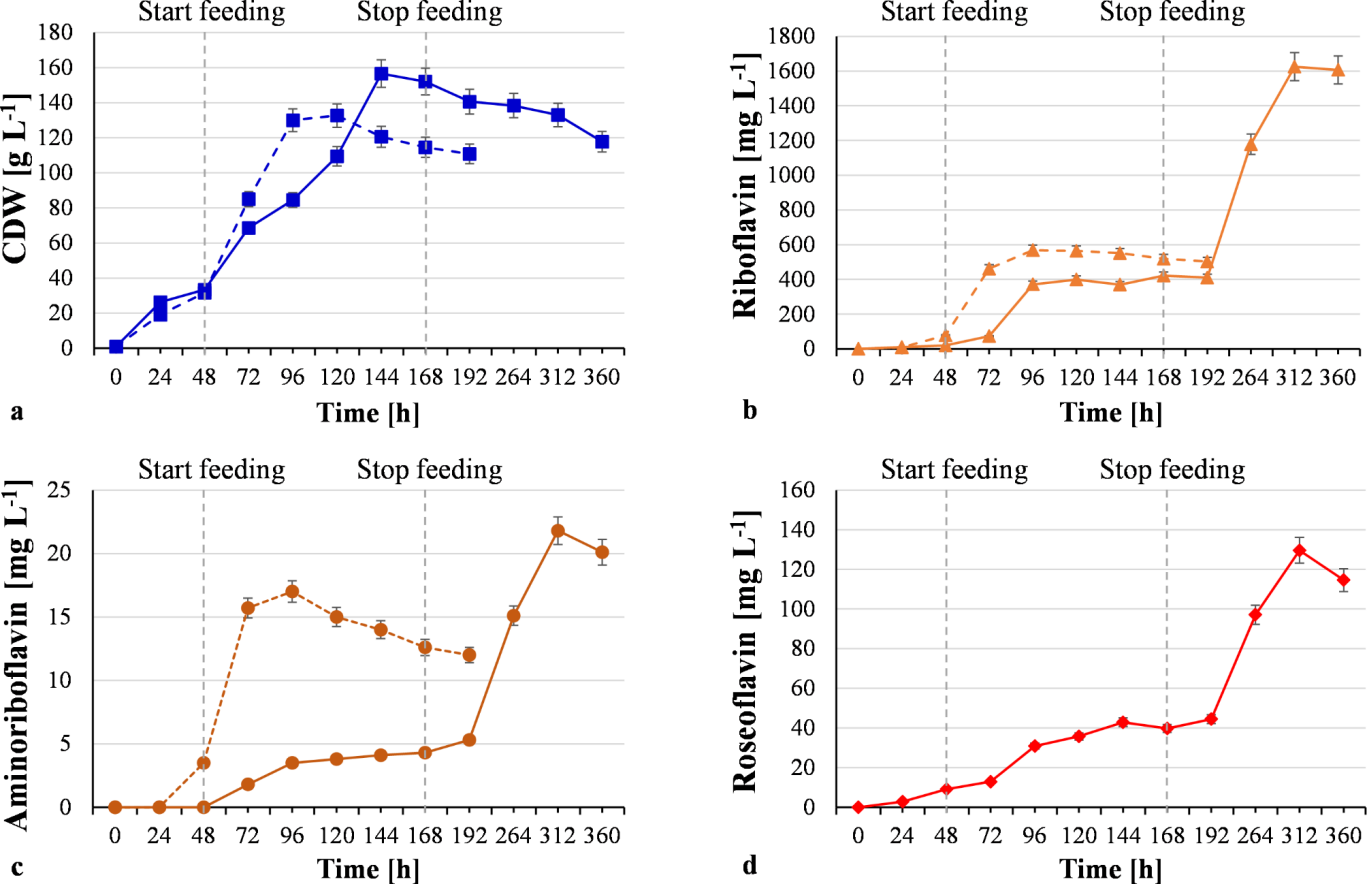
Growth **(a)**, riboflavin **(b)**, aminoriboflavin **(c)** and roseoflavin **(d)** production of engineered Y-33/FMN1-rosB (dash line) and Y-33/FMN1-rosB-rosA-rosC (solid lines) strains under conditions of high cell density cultivation in bioreactor

To assess the potential of the constructed strains, Y-33/FMN1-rosB and Y-33/FMN1-rosB-rosA-rosC, to produce flavin antibiotics, they were grown in fed-batch mode in bioreactors with glucose as the carbon source, Table 1; Fig. 4 showing the results of bioreactor cultivations.

Strain Y-33/FMN1-rosB accumulated 17 mg L^-1^ AF and 569 mg L^-1^ riboflavin after 96 h of fed-batch cultivation. AF and riboflavin were production during the exponential growth phase. After growth cessation, AF and riboflavin synthesis was also suspended. Riboflavin production by parental strain Y-33 was slower than that of Y-33/FMN1-rosB, reaching its highest titer 388 mg L^-1^ after 165 h cultivation Table 1. The data led us to conclude that AF stimulates riboflavin production.

Maximum riboflavin, AF and RoF production by strain Y-33/FMN1-rosB-rosA-rosC occurred after 312 h cultivation, amounting to 1625.4 mg L^-1^, 21.8 mg L^-1^ and 129.6 mg L^-1^, respectively. Moderate accumulation of flavins occurred during exponential growth phase from 48 to 144 h (Fig. 4). A sharp increase in production of flavins began after cessation of nutrient feeding. Intense accumulation of AF and RoF overlap exactly with increased riboflavin production, thereby confirming the hypothesis that riboflavin protects cells from the harmful effects of flavin antibiotics. Both strains produced negligible amounts of FMN (up to 1 mg L^-1^) (Table 1).

## Discussion

Our results show that yeasts are able to synthesize bacterial antibiotics after introduction of the corresponding genes. There are no data on the overproducers of the promising antibiotic AF. Herein we have described the construction and evaluation of *C. famata* and *K. phaffii* strains producing AF. Each organism was able to synthesize AF; however, *K. phaffii* synthesized ~ 1.3-fold more antibiotic in flasks compared to *C. famata* recombinant strains. High cell density cultivations of *K. phaffii* strain reached 17 mg AF L^− 1^ after 96 h. Purified AF had clear bacteriostatic and bactericidal activity against *S. aureus*, including MRSA, and *L. monocytogenes*. According to the literature, AF inhibits the growth of *L. monocytogenes* at a concentration of 100 mg L^− 1^ [16], which is 2-fold lower than the concentration used in our experiments. The effect of AF on *S. aureus* has not been previously studied. It is noteworthy that recombinant strains of *C. famata* and *K. phaffii* accumulated AF in the cultural media, but not AFP, despite the absence of bacterial *rosC* gene coding the specific AFP phosphatase [4]. Apparently, some of yeast endogenous unspecific phosphatases hydrolyzed AFP to AF. Growth of AF producing strain was not inhibited by the synthesized antibiotic during high cell density cultivations, at least at 17 mg L^− 1^.

Despite proper expression of *rosA* gene, we were unable to isolate RoF producer based on *C. famata*, the reason being the lack of activity of RosA. Although *C. famata* is a very active producer of riboflavin that acts as the precursor of flavin antibiotics, this organism is unsuitable for construction of RoF producer, apparently due to improper folding or some specific inhibitors of RosA in this yeast host. On the other hand, an efficient RoF producer was constructed using *K. phaffii.* Titer of RoF produced by recombinant *K. phaffii* (130 mg L^− 1^) was 9.3-fold higher than that obtained in the best recombinant native producer *S. davaonensis* (14 mg L^− 1^) [5]. However, when comparing strains cultivated in a batch culture with flasks, the production of RoF by engineered *S. davaonensis* exceeded that of recombinant *K. phaffii* by 2.8-fold. RoF at up to 40 mg L^− 1^ did not inhibit biomass accumulation during the course of high cell density cultivation. However, at higher concentration it impaired yeast growth. A constructed *K. phaffii* strain, in addition to RoF, synthesized a significant amount of AF as a byproduct. AF production by Y-33/FMN1-rosB-rosA-rosC was 1.3-fold higher than that by Y-33/FMN1-rosB, reaching 21.8 mg L^− 1^. Nevertheless, Y-33/FMN1-rosB-rosA-rosC had a 2.6-fold decrease in the AF production rate compared to strain Y-33/FMN1-rosB.

Synthesis of RoF is accompanied by a significant increase of riboflavin production. We were able to reach 1.6 g L^− 1^ riboflavin, which is highest titer described in non-patent literature so far for yeast, with exception of the flavinogenic yeasts, *C. famata* [40]. It is noteworthy activation of riboflavin, AF and RoF synthesis after cessation of cell feeding. It looks typical for the synthesis of secondary metabolites in the native producers; however, here we expressed *rosB, rosC* and *rosA* genes under the control of a constitutive promoter, and thus it was natural to get maximal synthesis of antibiotics during the growth phase. The phenomena were also quite unexpected because riboflavin, the primary metabolite, was also mostly synthesized after feeding was stopped. This phenomenon needs more substantial investigation in future. Apparently less or no toxic RoFMN and/or RoFAD are synthesized in the stationary phase due to cessation of primary metabolism; additionally, accumulated RoF does not inhibit cell growth as growth does not occur, hence cells better realize their biosynthetic potential. Kinetics of riboflavin synthesis is similar to that of RoF and AF as their synthesis starts to be activated only after drop of toxic RoFMN and RoFAD accumulation. It should be mentioned here that filamentous fungi *Ashbya gossypii* and *Eremothecium ashbyii* naturally overproduce riboflavin after growth cessation in the stationary phase [23].

Based on the accumulated data, one may assume that yeast make a promising platform for further development of efficient producers of flavin antibiotics. Further elevation of flavin antibiotic production could be achieved by an increase in riboflavin synthesis because this vitamin is the biosynthetic precursor of flavin antibiotics and since vitamin B2 could protect cells against the toxic action of RoF. The mitigation of the toxic effect of RoF and an increase of its production can be achieved through the expression in *K. phaffii* of riboflavin efflux protein homologous to that described in our previous work with *C. famata* [28]. Increase in RoF production can also be achieved by a decrease in AF accumulation via tuning the expression of the *rosA* gene. Adaptive laboratory evolution of the constructed strains towards increased resistance to RoF could further improve strain performance. The maximum titer of RoF was attained after 13 days of cultivation in the bioreactor. It can be speculated that such a relatively long process duration may have an impact on the viability and, consequently, the profitability of flavin antibiotics production by yeasts. Therefore, shortening the cultivation time poses a notable challenge, which can be overcome by adjusting the cultivation conditions and protocols employed within the bioreactor.

## Materials and methods

### Strains, media, cultivation conditions

*C. famata* VKMY-9 (wild type, from All-Russian Collection of Microorganisms, Pushchino, Russia), AF-4 [25], AF-4/FMN1/RIB1 (#18/13) (designated as FP from the FMN Producer) [29], AF-4/SEF1/RIB1/RIB7 (designated as BRP from the Best Riboflavin Producer) [26], *D. hansenii* CBS767 (wild-type laboratory strain), *Ogataea polymorpha* NCYC495 (wild -type laboratory strain) and *K. phaffii* X-33 (wild-type, from Invitrogen, Carlsbad, CA, USA), Y-33 (multi-copy overexpression of the riboflavin biosynthetic pathway) strains (Supplementary Table 1) were used throughout this work, and were grown at 30 °C on rich YPD (0.5% yeast extract, 1% peptone and 2% glucose), or mineral YNB (0.67% Yeast Nitrogen Base and 2% glucose) media. To estimate the synthesis of flavins, the yeast cells from a fresh plate were pre-grown in YPD for 2 days before cultures were inoculated with a starting OD_600_ of 5.0 in 50 mL YNB in 250 mL Erlenmeyer flasks and incubated during 72 h with shaking at 200 rpm at 30 °C. Applying a starting OD600 of 5.0 resulted in reproducible production of flavin antibiotics.

The *Escherichia coli* strain DH5α [Φ80d*lac*ZΔM15, *recA*1, *endA*1, *gyrA*96, *thi-*1, *hsdR*17(r^−^ _K_ m^+^ _K_), *supE*44, *relA*1, *deoR*, Δ(*lacZYA-argF*) U169] was used in experiments that required a bacterial host. DH5α was grown at 37 °C in LB medium as described [41]. Transformed *E. coli* cells were maintained in rich medium containing 100 mg L^− 1^ of ampicillin or 50 mg L^− 1^ of NTC.

Strains of *S. aureus* ATCC 25,923 and MRSA ATCC 43,300 and *L. monocytogenes* ATCC 19,113 were cultivated in 5 mL Tryptic Soy Broth (TSB) in 15 mL test tubes with shaking at 200 rpm at 37 °C. AF was added to the growth media at a final concentration of 200 mg L^− 1^. Growth was estimated by counting bacterial colonies on Tryptic Soy Agar plates incubated for 18 h at 37 °C.

### Molecular-biology techniques

Standard cloning techniques were used as described by [41]. Genomic DNA of yeasts was isolated using the NucleoSpin® Tissue Kit (Macherey-Nagel, Duren, Germany). Restriction endonucleases and DNA ligase (Thermo Fisher Scientific Baltics, Vilnius, Lithuania) were used. Plasmid isolation from *E. coli* involved the Zyppy™ Plasmid Miniprep (Irvine, CA, USA). PCR-amplification of the fragments of interest was achieved with Phusion High-Fidelity DNA Polymerase (Thermo Fisher Scientific Baltics, Vilnius, Lithuania). PCRs were obtained using a GeneAmp PCR System 9700 thermocycler (Applied Biosystems, Foster City, CA, USA). The PCR thermocycling conditions were employed according to the manufacturer’s instructions. The Thermo Fisher Tm calculator was utilized to estimate the suitable annealing temperature. (https://www.thermofisher.com/ua/en/home/brands/thermo-scientific/molecular-biology/molecular-biology-learning-center/molecular-biology-resource-library/thermo-scientific-web-tools/tm-calculator.html).

### Plasmids construction

For overexpression of *rosB* from *S. davaonensis* in yeast *C. famata*, a recombinant plasmid was constructed on the basis of the plasmid pTTb [28]. A codon optimized version of *rosB* (Supplementary Sequences) was synthetized based on NCBI Gene ID 31,229,912 by BioCat GmbH (Heidelberg, Germany), before being BamHI/PstI-digested and subcloned into corresponding sites of plasmid pTTb. The *IMH3* gene encoding inosine monophosphate dehydrogenase conferring resistance to mycophenolic acid [25] was PCR-amplified from genomic DNA of *D. hansenii* CBS767, using a pair of primers IMH3F (sequences of all used primers are shown in Supplementary Table 3) and IMH3R and cloned into the XhoI site of the former plasmid. XhoI restriction sites were introduced into the primers to simplify cloning. The resulting plasmid was designated as prosB-IMH3 (Supplementary Fig. 3a).

Construction of the plasmid for simultaneous overexpression of the *FMN1, rosB, rosA* and *rosC* genes in *C. famata* involved in several steps. Similar to *rosB*, optimized *rosA* from *S. davaonensis* (Supplementary Sequences) was synthetized, based on NCBI Gene ID 31,229,948 and then BamHI/PstI-digested, before being subcloned into corresponding sites of plasmid pTTb to create prosA. Then the promoter of the *TEF1* gene of *C. famata* and the *rosA* gene together with the terminator of the *TEF1 D. hansenii* were amplified by PCR using primer pairs Ko833 / Ko834 and Ko835 / Ko836, and genomic DNA of *C. famata* VKMY-9 and prosA as templates, respectively. Both fragments were combined by a PCR using primers Ko833/ Ko836 and cloned into the KpnI site of the pUC57 to create pUC57-rosA. At the next stage, the promoter of the *TEF1* of *C. famata* and the *rosB* together with the terminator of the *TEF1 D. hansenii* were amplified by PCR using primer pairs Ko827 / Ko828 and Ko829 / Ko830, respectively. Genomic DNA of *C. famata* VKMY-9 and prosB acted as templates. Both fragments were combined by overleap PCR using a pair of primers Ko827 / Ko830. PCR fragment containing pUC57 and the expression module of *rosA* was amplified from pUC57-rosA with primers Ko833 / Ko838. The expression module containing the *D. hansenii FMN1* gene under the control of the *TEF1Cf* promoter and its own terminator was amplified from the vector p19L2_ble_RIB1Cf_prTEF1_FMN1Dh [29] using a pair of primers Ko825 / Ko826. The *IMH3* gene was amplified from *D. hansenii* CBS767 genomic DNA using a pair of primers Ko831 / Ko832. Four fragments containing the expression modules for *rosA*, *rosB*, *FMN1* and *IMH3* were used to combine into single plasmid using the Gibson Assembly. The resulting plasmids were designed as pFMN1-rosB-rosA-IMH3 (Supplementary Fig. 4a).

To use an alternative yeast selectable marker, the gene *SAT-1 E. coli* conferring resistance to NTC was PCR amplified from plasmid pTb/SAT-1 [42] using a pair of primers Ko1062 / Ko1063. The fragment was cloned into the ApaI site of the plasmid pFMN1-rosB-rosA-IMH3 instead of *IMH3*, resulting in pFMN1-rosB-rosA-SAT (Supplementary Fig. 4b).

Optimized *rosC S. davaonensis* (Supplementary Sequences) was synthetized based on NCBI Gene BN159_8033 and amplified with primers Ko1075 / Ko1076, BamHI/PstI-digested and subcloned into pUC57-rosA instead of *rosA* to create pUC57-rosC. The expression module of *rosC* was amplified with a pair of primers Ko1077 / Ko1078 and pUC57-rosC as a template and cloned into the EcoRI site of the plasmid pFMN1-rosB-rosA-SAT, resulting in pFMN1-rosB-rosA-rosC-SAT (Supplementary Fig. 4c).

Construction of recombinant plasmids for expression of target genes in *K. phaffii* was done using the GoldenPiCS system based on Golden Gate cloning [43]. ORF *FMN1 K. рhaffii* was amplified by PCR from genomic DNA of *K. рhaffii* X-33 with a pair of primers BsaI_FS2_FMN1_Pipa_FW / BsaI_FS3_FMN1_Pipa_BW. Then *FMN1* was cloned between the *TEF1* promoter and *IDP1* terminator to create BB2-BC-TEF1-FMN1-IDP1. The *rosB S. davaonensis* gene was amplified with a pair of primers, BsaI_FS2_rosB_syn_FW / BsaI_FS3_rosB_syn_BW, and vector pPICZA-rosB containing the *rosB* gene codon optimized for *K. рhaffii* (Supplementary Sequences) as a template. *rosВ* was cloned between the *GAP1* promoter and *RPS2* terminator to create BB2-AB-GAP1-rosB-RPS2. At the next stage, both the expression modules were combined in a single plasmid containing *RGI2* as a locus for plasmid integration and selective marker gene *natNT2* for selection of yeast transformants on NTC-containing medium. This plasmid was named pFMN1-rosB-NAT (Supplementary Fig. 5a).

The same approach was used for construction of a plasmid containing 3 expression modules for *FMN1*, *rosB* and *rosА*. *rosA* was amplified with a pair of primers BsaI_FS2_rosA_syn_FW / BsaI_FS3_rosA_syn_BW, and with vector pPICZA-rosA containing codon optimized *rosA* gene (Supplementary Sequences) as a template. Then *rosA* was cloned between the *GAP1* promoter and *RPS2* terminator to create BB2-CD-GAP1-rosA-RPS2. The expression modules for *FMN1*, *rosB* and *rosА* in part with a fragment containing a non-transcribed intergenic spacer (NTS) of rDNA locus for multicopy integration were combined in a single plasmid containing selective marker gene *natNT2*. A unique restriction site for AscI was introduced into the NTS with a pair of primers Ko1035 / Ko1036 and the former plasmid as a template. The constructed plasmid was named pFMN1-rosB-rosA-NAT (Supplementary Fig. 6a).

*rosC* was amplified with a pair of primers Ko1081 / Ko1082 and vector pUC57-rosC containing codon optimized ORF of *rosC* gene (Supplementary Sequences) as a template. PCR fragment containing vector and *GAP1* promoter and *RPS2* terminator was amplified with a pair of primers Ko1083 / Ko1084 and BB2-CD-GAP1-rosA-RPS2 as a template. Both fragments were combined into a single plasmid using the Gibson Assembly to create BB2-CD-GAP1-rosC-RPS2. The recipient strain of *K. phaffii* able to RoF production does not contain free selective markers. To overcome this drawback, additional selective markers needed to be applied. *SUC2 S. cerevisiae* encoding invertase can be used as a marker for selection of transformants on a medium containing sucrose [44]. The expression module of *rosC*, gene *SUC2 S. cerevisiae*, NTS and vector pUC57 were amplified with a pairs of primers Ko1085 / Ko1086, Ko1087 / Ko1088, Ko1089 / Ko1090 and Ko1091 / Ko1092, respectively. Four fragments were combined together using Gibson Assembly to create prosC-SUC2.

Gene *IMH3* was used in our previous work as a marker for selection of *C. famata* and *O. polymorpha* transformants [45]. The gene was amplified from genomic DNA of *O. polymorpha* NCYC495 with a pair of primers Ko1140 / Ko1141. The vector was amplified with primers Ko1142 and Ko1143, with plasmid prosC-SUC2 as a template. Both fragments were combined using Gibson Assembly to create prosC-IMH3.

Gene *BSD* was used as a marker for the selection of *K. phaffii* transformants on the medium containing antibiotic blasticidin [46]. The gene *BSD* under control of *TEF1 D. hansenii* promotor and terminator was amplified with primers Ko1147 and Ko430 and plasmid pUC57/prTEF1_BSD (42) as a template. The vector containing expression module of *rosC* was amplified with a pair of primers Ko1145 / Ko1146 and plasmid prosC-SUC2 as a template. Both fragments were digested by XbaI and SalI and ligated to create plasmid prosC-BSD (Supplementary Fig. 6c).

The accuracy of the constructed plasmids was verified by sequencing.

### Strains construction

*C. famata* strain FP overproducing FMN [29] was used as the parental for overexpression of *rosB*. The plasmid prosB-IMH3 was linearized with the restriction endonuclease AatII and used for transformation of strain FP. The transformants were selected on a solid mineral medium, containing mycophenolic acid at 15–20 mg L^-1^ after one week of incubation. The selected transformants in this and subsequent cases were stabilized by alternating cultivation on a non-selective medium for 15–20 generations followed by selective media. Subsequently, the selected strain FP/rosB (Supplementary Table 1) was checked with PCR using a pair of primers RBFa / RBRa to verify of presence the expression module of *rosB* (Supplementary Fig. 3b).

*C. famata* strain BRP/FMN1-rosB-rosA-rosC (Supplementary Table 1) expressing *FMN1, rosB, rosA* and *rosC* genes was constructed based on BRP after its transformation with a AatII-linearized plasmid pFMN1-rosB-rosA-rosC-SAT. Selective YPD medium contained 20 mg L^− 1^ of NTC. The presence of the corresponding expression modules in the genome of constructed strain was confirmed by PCR, using primers Ko1058, Ko1059, Ko1060, Ko1061 for modules *FMN1, rosB, rosA* and a pair of primers Ko1058 / Ko1079 for *rosC* (Supplementary Fig. 4d, Supplementary Fig. 4e).

The AF producing strain of *K. phaffii* was constructed by transformation of Y-33 with AscI-linearized plasmid pFMN1-rosB-NAT, with subsequent selection on YPD with 100 mg L^− 1^ NTC. The selected strain Y-33/FMN1-rosB (Supplementary Table 1) was checked with PCR using pairs of primers Ko1037 / Ko1038 and Ko1039 / Ko1040 to confirm the presence of the expression modules *FMN1* and *rosB*, respectively (Supplementary Fig. 5b).

The RoF producing strain of *K. phaffii* was constructed by transformation of Y-33 with AscI-linearized plasmid pFMN1-rosB-rosA-NAT with selection on YPD with 100 mg L^− 1^ of NTC. To achieve the multicopy integration, the transformants were transferred to YPD agar plates by stepwise increasing the concentration of NTC from 100 mg to 4000 mg L^− 1^, finally gaining the strain Y-33/FMN1-rosB-rosA (Supplementary Table 1). This strain was verified by PCR using pairs of primers Ko1037 / Ko1038, Ko1039 / Ko1040 and Ko1037 / Ko1042 to confirm the presence of the expression modules *FMN1, rosB* and *rosA*, respectively (Supplementary Fig. 6b).

To increase RoF production, AscI-linearized plasmids prosC-SUC2, prosC-IMH3 or prosC-BSD were transformed to the Y-33/FMN1-rosB-rosA. We could select transformants only on the medium supplemented with blasticidin, at 500 mg L^− 1^. Finally, the selected strain Y-33/FMN1-rosB-rosA-rosC (Supplementary Table 1) was verified by PCR using pairs of primers Ko1037 / Ko1093 to confirm the presence of the expression module of *rosC* (Supplementary Fig. 6d).

### Quantitative real-time PCR

Expression of the *FMN1*, *rosB*, *rosA* and *rosC* genes was confirmed by qRT-PCR. Total RNA was extracted from yeast cells using the GeneMATRIX Universal RNA Purification Kit with DNAseI (EURx Ltd., Gdansk, Poland). qRT-PCR involved the 7500 Fast Real-Time PCR System (The Applied Biosystems, USA), with SG OneStep qRT-PCR kit (EURx Ltd., Gdansk, Poland) using corresponding pairs of primer (Supplementary Table 3), RNA as a template and ROX reference passive dye previously described [27]. The expression of the *FAD1* gene in *C. famata* and *K. phaffii* was used as a normalization reference for the *rosA, rosB*, and *rosC* genes in the engineered strains.

### Fed-batch fermentations

A preculture of the respective *K. phaffii* strains incubated with shaking at 28 °C for 24 h on YPG (per liter: 10 g yeast extract, 10 g peptone, 10 g glycerol) was used to inoculate the starting volume (400 mL batch medium) of the bioreactors to a starting optical density at 600 nm of 5.0. Fermentations were carried out in 1 L working volume bioreactors with a computer-based process control (BioFlo 120, Eppendorf, Germany). Fermentation temperature was controlled at 25 °C, pH at 5.0, with addition of 12.5% ammonium hydroxide, and the dissolved-oxygen concentration being maintained above 20% saturation by controlling the stirrer speed between 600 and 1,200 rpm.

The batch medium contained (per L) 2.0 g citric acid, 12.4 g (NH_4_)_2_HPO_4_, 0.022 g CaCl_2_·2H_2_O, 0.9 g KCl, 0.5 g MgSO_4_·7H_2_O, 46.5 g glycerol, and 4.6 mL PTM_1_ trace salts stock solution. The pH was adjusted to 5.0 with 25% HCl. Glucose fed-batch solution contained (per L) 550 g glucose·1 H_2_O, 10 g KCl, 6.45 g MgSO_4_·7H_2_O, 0.35 g CaCl_2_·2H_2_O, and 12 mL PTM_1_ trace salts stock solution. The PTM_1_ trace salts stock solution contained (per L) 6.0 g CuSO_4_·5H_2_O, 0.08 g NaI, 3.0 g MnSO_4_·H_2_O, 0.2 g Na_2_MoO_4_·2H_2_O, 0.02 g H_3_BO_3_, 0.5 g CoCl_2_, 20.0 g ZnCl_2_, 65.0 g FeSO_4_·7H_2_O, 0.2 g biotin, and 5.0 mL H_2_SO_4_ (95 to 98%).

After 48 h, the batch was completed and the glucose fed-batch (a feed rate of 3.6 g h^–1^) was started for ~ 120 h. Samples were taken once per day. Flavins concentrations were determined by HPLC.

### N,N-8-Amino-8-demethyl-D-riboflavin dimethyltransferase assay

N,N-8-Amino-8-demethyl-D-riboflavin dimethyltransferase (RosA) activity was measured as has been described elsewhere, with slight modifications [37]. To determine the specific activity of RosA, yeast cells were grown to mid-log phase on YNB and cell-free extracts were prepared by using *glass beads*. Protein concentration was determined by the Lowry method [47]. The activity of RosA was determined in a reaction mixture of the following composition (1 mL): 50 mM Tris-HCl, pH 8.0; 5 mM AF; 2 mM S-adenosyl methionine (SAM) and a cell-free extract at 0.5 mg mL^− 1^. The mixture was preincubated at 37 °C for 5 min, and the reaction was started by the addition of SAM. After 90 min incubation at 37 °C, an aliquot was applied to an HPLC column. RosA activity was expressed as µmol of RoF formed min^− 1^ from AF and SAM.

### AF and RoF purification

AF and RoF purification was carried out in 2 stages. The supernatant fluid was passed through a column of magnesium silicate (Florisil® Adsorbent 60–100 Mesh) (diameter, 1.4 cm; height, 20 cm; flow rate, 100 mL h^− 1^). The column was washed with water (500 mL) to remove unbound compounds. Fluorescent material was then eluted with 50% acetone: H_2_O (2 : 1, v/v). Fractions with a volume 3 mL were collected and spectrally analyzed. The flavin containing fractions obtained in the previous step were applied to a column of cellulose for chromatography (diameter, 2.4 cm; height, 50 cm). A column with cellulose was washed with 300 mL water; 3–4 mL of concentrated flavin fractions were loaded on the column. Flavins were eluted with water (flow-rate 30 mL h^− 1^). Fluorescent or visible red fractions were collected. Purified AF and RoF were identified by spectral analysis, mass spectrometry and chromatographic methods (Fig. 2; Supplementary Fig. 1; Supplementary Fig. 2). The yield of isolated AF was 35%, while that of RoF was 25%.

### Chromatographic analysis

Flavins concentrations were determined by high performance liquid chromatography (HPLC) using a Sigma Nucleosil C18 (10 mm × 4.6 mm ID, 5 μm) guard column and a Sigma Nucleosil C18 (150 mm × 4.6 mm ID, 5 μm) column or Ascentis^®^ C18 Supelguard^™^ (20 mm × 4 mm ID, 5 μm) and Ascentis^®^ C18 (250 mm × 4.6 mm ID, 5 μm) with an isocratic flow at 1 mL min^-1^ running buffer (50 mM NaH_2_PO_4_-H_3_PO_4_ pH3; 1 mM tetramethyl ammonium chloride; 12% acetonitrile (v/v)) [48].

As the solubility of riboflavin is ~ 200 mg L^-1^, a 1 mL aliquot of culture broth was diluted to adjust the riboflavin content of the mixture to < 200 mg L^-1^. The mixture was autoclaved to dissolve remaining crystals of riboflavin. Supernatant was obtained by centrifugation in 2 mL tubes for 1 min at 13,000 rpm. Prior to injection on the HPLC column, the samples were mixed with a 2-times concentrated running buffer in an equal ratio and filtered over PVDF Durapore Syringe Driven Filter Unit (Millipore) with a pore size of 0.22 μm. 100 µL of the final samples were loaded on the HPLC column and quantified by the peak height of absorption, detected at 223 and 445 nm respectively.

Ultra-high performance liquid chromatography coupled mass spectrometry (UHPLC-MS) was carried out on an ACQUITY system and Synapt G2 mass spectrometer with hybrid Quadrupole and Time-of-flight analyzer (Waters Corp., Milford, MA, USA). The separation of flavins in the purified product of yeast cells was achieved using ACQUITY UPLC BEH C18 Column, 130Å, 1.7 μm, 2.1 mm × 100 mm column (Waters, USA). The column temperature was set at 40 °C, whereas the autosampler was maintained at 4 °C, the injection volume being 5 µL. The mobile phases for UHPLC-MS were solvent A (10 mM ammonium formate and 0.1% formic acid in water) and solvent B (10 mM ammonium formate and 0.1% formic acid in methanol) at a flow-rate 0.35 mL min^− 1^. The linear gradient elution was used as follows: 0 min, 5% B; 10 min, 90% B; 12 min, 90% B; back to 5% in 0.1 min. A 1.9 min equilibration time was used between injections. Analysis was made using electrospray ionization in positive ion mode (ES+). The optimized MS parameters were as follows: source temperature of 350 °C, desolvation temperature 120 °C, cone gas flow 50 L h^− 1^, desolvation gas flow 550 L h^− 1^, capillary voltage of 3.6 kV, and cone voltage of 30 V. MS analysis used a scan range from 150 to 900 m/z. MassLynx 4.1 software (Waters, USA) was used for instrument control and data analysis.

HPLC-DAD separation was carried out using Dionex system, equipped with a quaternary pump, diode array detector, autosampler and degasser (Dionex UltiMate 3000, Thermo Fisher Scientific, Waltham, MA, USA). ACE C18 (4.6 × 250 mm, particle size 5 μm) column was used at 40 °C. The mobile phase consisted of solvent A (10 mM ammonium formate and 0.1% formic acid in water) and solvent B (10 mM ammonium formate and 0.1% formic acid in methanol) at a flow rate 1 mL min^− 1^. For separation of the AF, the linear gradient elution was used as follows: 0 min, 5% B; 5 min, 90% B; 6 min, 90% B; and back to 5% in 0.2 min. A 3.8 min equilibration time was used between injections. The injection volume was maintained at 20 µL. For separation of the RoF, the linear gradient elution was used as follows: 0 min, 5% B; 1 min, 35% B; 10 min, 35% B; 10.5 min, 95% B; 12.5 min, 95% B; and back to 5% in 0.5 min. A 1.0 min equilibration time was used between injections. The injection volumes were maintained at 30–50 µL. RoF was detected at 509 nm and identification was specified on the basis of retention time and UV-Vis spectra of commercial standard (Sigma-Aldrich). Dionex software (Chromeleon ver. 7.8) was used for collection and processing of data analysis. UV-VIS spectra were recorded at the range of 210–600 nm.

### Mass spectrometry

Samples were measured on an Amazon ETD Ion Trap Mass Spectrometer (Bruker, Billerica, MA, USA) after injection into the mass spectrometer by direct infusion in positive and negative electrospray mode. Nitrogen was used for both drying and nebulizing. The temperature of the drying gas in the ionization source was 220 °C. Gas flow was 5 L min^− 1^, the nebulizer pressure 10 psi, and the capillary voltage 4,500 V. After the MS experiment, MS/MS was analyzed for the ion of interest. Furthermore, fragmentation analysis MS^3^ was carried out for the important ion obtained after MS^2^. Data were processed using Data Analysis 4.2 software (Bruker Daltonics) software.

### Statistical analysis

All the experimental data shown in this manuscript were collected from three independent samples to ensure reproducibility of the trends and relationships observed in the cultures. Each error bar indicates the standard deviation (SD) from the mean obtained from triplicate samples. The 5% significance level was used in the statistical analyses. Unless otherwise specified, all cultivation experiments were conducted in triplicate or more.

## Electronic supplementary material

Below is the link to the electronic supplementary material.

**Additional file 1: Figure S1.** Identification of aminoriboflavin by HPLC-DAD. **Figure S2.** Chromatographic separation of roseoflavin by HPLC-DAD. **Figure S3.** (a) Linear scheme of plasmid prosB-IMH3, (b) PCR verification of FP/rosB strain. **Figure S4.** Linear schemes of plasmids (a) pFMN1-rosB-rosA-IMH3, (b) pFMN1-rosB-rosA-SAT, (c) pFMN1-rosB-rosA-rosC-SAT, (d) PCR verification of BRP/FMN1-rosB-rosA-rosC strain, (e) PCR verification of BRP/FMN1-rosB-rosA-rosC strain. **Figure S5.** (a) Linear scheme of plasmid pFMN1-rosB-NAT, (b) PCR verification of Y-33/FMN1-rosB strain. **Figure S6.** (a) Linear scheme of plasmid pFMN1-rosB-rosA-NAT, (b) PCR verification of Y-33/FMN1-rosB-rosA strain, (c) Linear scheme of plasmid prosC-BSD, (d) PCR verification of Y-33/FMN1-rosB-rosA-rosC strain. **Table S1.** Strains used in this study. **Table S2.** Relative expression levels of *FMN1, rosB, rosA* and *rosC* genes in FP/rosB, BRP/FMN1-rosB-rosA-rosC, Y-33/FMN1-rosB and Y-33/FMN1-rosB-rosA-rosC strains versus the recipient strains AF-4, BRP and Y-33. **Table S3.** List of primers used in this study. **Sequences S.** Sequences of genes *rosB, rosC, rosA* optimized for *C. famata* and *K. phaffii*

## Data Availability

All data are available in the main text or the supplementary materials.

## Abbreviations

AF: Aminoriboflavin or 8-demethyl-8-aminoriboflavin
AFP: Aminoriboflavin-5’-phosphate or 8-demethyl-8-aminoriboflavin-5’-phosphate
BRP: Best riboflavin producer
CDW: Cell dry weight
ESI-MS: Electrospray Ionisation Mass Spectrometry
FMN: Flavin mononucleotide
HPLC-DAD: High-performance liquid chromatography with photodiode-array detection
Lm: *Listeria monocytogenes*
MRSA: Methicillin-resistant Staphylococcus aureus
NTC: Nourseothricin
RoF: Roseoflavin or 7-methyl-8-dimethylamino-(1’-D-ribityl)isoalloxazine)
Sa: *Staphylococcus aureus*
