# Supplementary material for: Efficient production of bacterial antibiotics aminoriboflavin and roseoflavin in eukaryotic microorganisms, yeasts
Source: Microb Cell Fact. 2023 Jul 20;22:132. doi: 10.1186/s12934-023-02129-8 (PMC10357625; doi:10.1186/s12934-023-02129-8)
Supplement: Supplementary file 1 — Additional file 1: Figure S1. Identification of aminoriboflavin by HPLC-DAD. Figure S2. Chromatographic separation of roseoflavin by HPLC-DAD. Figure S3. (a) Linear scheme of plasmid prosB-IMH3, (b) PCR verification of FP/rosB strain. Figure S4. Linear schemes of plasmids (a) pFMN1-rosB-rosA-IMH3, (b) pFMN1-rosB-rosA-SAT, (c) pFMN1-rosB-rosA-rosC-SAT, (d) PCR verification of BRP/FMN1-rosB-rosA-rosC strain, (e) PCR verification of BRP/FMN1-rosB-rosA-rosC strain. Figure S5. (a) Linear scheme of plasmid pFMN1-rosB-NAT, (b) PCR verification of Y-33/FMN1-rosB strain. Figure S6. (a) Linear scheme of plasmid pFMN1-rosB-rosA-NAT, (b) PCR verification of Y-33/FMN1-rosB-rosA strain, (c) Linear scheme of plasmid prosC-BSD, (d) PCR verification of Y-33/FMN1-rosB-rosA-rosC strain. Table S1. Strains used in this study. Table S2. Relative expression levels of FMN1, rosB, rosA and rosC genes in FP/rosB, BRP/FMN1-rosB-rosA-rosC, Y-33/FMN1-rosB and Y-33/FMN1-rosB-rosA-rosC strains versus the recipient strains AF-4, BRP and Y-33. Table S3. List of primers used in this study. Sequences S. Sequences of genes rosB, rosC, rosA optimized for C. famata and K. phaffii [file 12934_2023_2129_MOESM1_ESM.docx]

**Microbial Cell Factories**

Supplementary file

**Efficient production of bacterial antibiotics aminoriboflavin and roseoflavin in eukaryotic microorganisms, yeasts**

**Authors**

Kostyantyn V. Dmytruk,^1#^ Justyna Ruchala,^2#^ Liubov R. Fayura,^1^ Grzegorz Chrzanowski,^2^ Olena V. Dmytruk,^1^, Andriy O. Tsyrulnyk,^1^ Yuliia A. Andreieva,^1^ Daria V. Fedorovych,^1^ Olena I. Motyka,^3^ Diethard Mattanovich,^4^ Hans Marx,^4^ Andriy A. Sibirny,^1,2^*

**Affiliations**

^1^ Institute of Cell Biology National Academy of Sciences of Ukraine, Drahomanov St, 14/16, Lviv, 79005, Ukraine.

^2^ University of Rzeszow, Zelwerowicza 4, 35-601 Rzeszow, Poland.

^3^ Research Institute of Epidemiology and Hygiene of the Danylo Halytsky Lviv National Medical University, Zelena St, 12, 79005, Lviv, Ukraine.

^4^ University of Natural Resources and Life Sciences, Vienna, Department of Biotechnology, Institute of Microbiology and Microbial Biotechnology, Muthgasse 18, 1190 Vienna, Austria.

^#^Equal contribution

*Corresponding author. Email: [sibirny@yahoo.com](mailto:sibirny@yahoo.com)

| **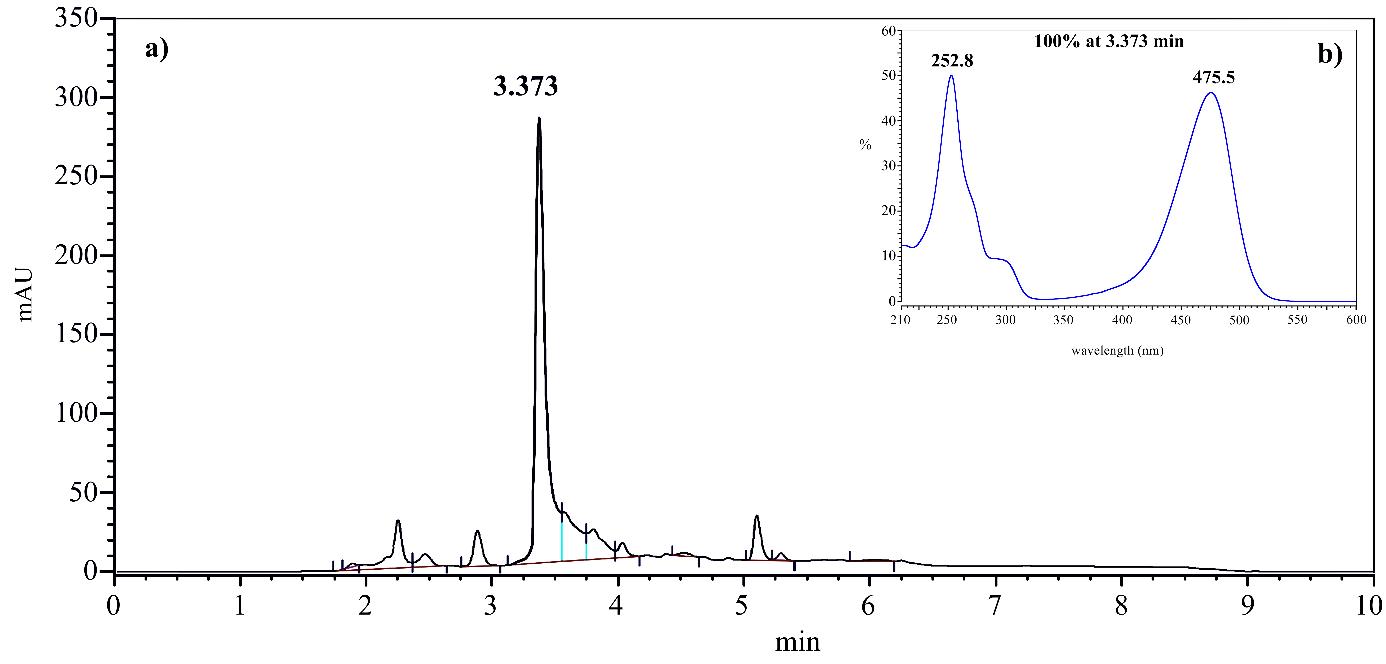** |
| --- |
| **Supplementary Figure 1**  Identification of aminoriboflavin by HPLC-DAD; a) chromatogram of aminoriboflavin separation, b) UV-VIS spectrum recorded for separated aminoriboflavin. |

| **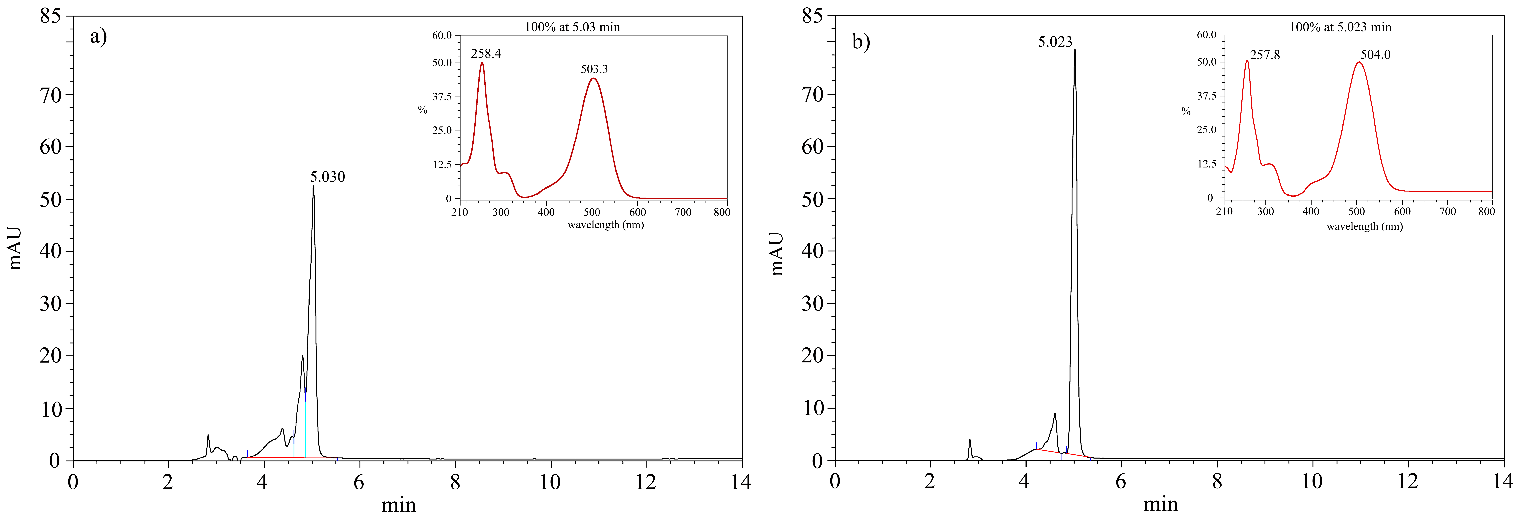** |
| --- |
| **Supplementary Figure 2**  Chromatographic separation of roseoflavin by HPLC-DAD; a) chromatogram and UV-VIS spectrum for roseoflavin from yeast cultivation, b) chromatogram and UV-VIS spectrum recorded for roseoflavin standard (Sigma-Aldrich). |

|  |
| --- |
| **Supplementary Figure 3** |
| (**a**) **Linear scheme of plasmid prosB-IMH3**. Promoter and terminator of the *TEF1 D. hansenii* are indicated as blue boxes; *rosB* – as orange box; selective markers *IMH3* and *ble*– as black boxes. (**b**) **PCR verification of FP/rosB strain** with pair of primers RBFa/RBRa, which amplify 753 bp fragment (positive and negative controls indicated as + and − ; 1-4 - FP/rosB and L—1 kb ladder). |

|  |
| --- |
| **Supplementary Figure 4** |
| **Linear schemes of plasmids** (**a**) **pFMN1-rosB-rosA-IMH3,** (**b**) **pFMN1-rosB-rosA-SAT and (c) pFMN1-rosB-rosA-rosC-SAT**. Promoter of the *TEF1* gene of *C. famata* and terminator of the *TEF1 D. hansenii* are indicated as blue boxes; *FMN1* – as yellow boxes; *rosB,* *rosA* and *rosC* – as orange, red and dark gray boxes, respectively; selective markers *IMH3* and *SAT-1* – as black boxes. (**d**) **PCR verification of BRP/FMN1-rosB-rosA-rosC** **strain** with pair of primers Ko1058 / Ko1059, which amplify 407 bp fragment; Ko1058 / Ko1060 – 691 bp and Ko1058 / Ko1061 – 1007 bp (positive and negative controls indicated as + and − ; 5 and 9 - BRP/FMN1-rosB-rosA-rosC and L—1 kb ladder). (**e**) **PCR verification of BRP/FMN1-rosB-rosA-rosC** **strain** with pair of primers Ko1058 / Ko1079, which amplify 310 bp fragment (positive and negative controls indicated as + and − ; 5 and 9 - BRP/FMN1-rosB-rosA-rosC and L—1 kb ladder). |

|  |
| --- |
| **Supplementary Figure 5** |
| (**a**) **Linear scheme of plasmid pFMN1-rosB-NAT**. Promoters of the *GAP*, *TEF1* and terminators of the *RPS2, IDP1* of *K. phaffii* are indicated as green boxes; *FMN1* – as yellow box; *rosB* – as orange box; selective marker *NAT* – as black box. (**b**) **PCR verification of Y-33/FMN1-rosB** **strain** with pair of primers Ko1037 / Ko1038, which amplify 865 bp fragment and Ko1039 / Ko1040 – 648 bp (positive and negative controls indicated as + and − ; 1 and 2 - Y-33/FMN1-rosB and L—1 kb ladder). |

|  |
| --- |
| **Supplementary Figure 6** |
| (**a**) **Linear scheme of plasmid pFMN1-rosB-rosA-NAT**. Promoters of the *GAP*, *TEF1* and terminators of the *RPS2, IDP1* of *K. phaffii* are indicated as green boxes; *FMN1, rosB* and *rosA* – as yellow, orange and red boxes, respectively; *NTS* – as pink box; selective marker *NAT* – as black box. (**b**) **PCR verification of Y-33/FMN1-rosB-rosA** **strain** with pair of primers Ko1037 / Ko1038, which amplify 865 bp fragment; Ko1039 / Ko1040 – 648 bp and Ko1037 / Ko1042 – 1126 bp (positive and negative controls indicated as + and − ; 1, 3, 4, 9, 11, 16-18 - Y-33/FMN1-rosB-rosA and L—1 kb ladder). (**c**) **Linear scheme of plasmid prosC-BSD**. Promoter of the *GAP* and terminator of the *RPS2* of *K. phaffii* are indicated as green boxes; *rosC* – as dark gray box; *NTS* – as pink box; selective marker *pTEF1-BSD* – as black box. (**d**) **PCR verification of Y-33/FMN1-rosB-rosA-rosC** **strain** with pair of primers Ko1037 / Ko1093, which amplify 403 bp fragment (positive control indicated as + ; 1-13 - Y-33/FMN1-rosB-rosA-rosC and L—1 kb ladder). |

**Supplementary Tables**

***Supplementary Table 1 Strains used in this study***

| **Strain name** | **Description** | **Reference** |
| --- | --- | --- |
| ***Debaryomyces hansenii*** |  |  |
| CBS767 | wt |  |
| ***Candida famata*** |  |  |
| VKMY-9 | wt |  |
| AF-4 | riboflavin overproducer isolated by conventional mutagenesis and classical selection from VKMY-9 | [25] |
| FP | AF-4 + *RIB1Cf* + P_TEF1Cf_-*FMN1Dh ble* | [29] |
| FP/rosB | FP + P*_TEF1Dh_*-*rosB*-*T_TEF1Dh_* *IMH3* | Present study |
| BRP | AF-4 + *RIB1Cf + RIB7Cf + SEF1Dh ARO4m* | [26] |
| BRP/FMN1-rosB-rosA-rosC | BRP + P_TEF1Cf_-*FMN1Dh* P*_TEF1Cf_*-*rosB*-*T_TEF1Dh_* P*_TEF1Cf_*-*rosA*-*T_TEF1Dh_* P*_TEF1Cf_*-*rosC*-*T_TEF1Dh_ SAT* | Present study |
| ***Komagataella*** ***phaffii*** |  |  |
| X-33 | wt |  |
| Y-33 | X-33 + multi-copy overexpression of the riboflavin biosynthetic pathway | Present study |
| Y-33/FMN1-rosB | Y-33 + P*_TEF1Kp_*-*FMN1*-*T_IDP1Kp_* P*_GAPKp_*-*rosB*-*T_RPS2Kp_ NAT* | Present study |
| Y-33/FMN1-rosB-rosA | Y-33 + P*_TEF1Kp_*-*FMN1*-*T_IDP1Kp_* P*_GAPKp_*-*rosB*-*T_RPS2Kp_* P*_GAPKp_*-*rosA*-*T_RPS2Kp_ NAT* | Present study |
| Y-33/FMN1-rosB-rosA-rosC | Y-33/FMN1-rosB-rosA + P*_GAPKp_*-*rosC*-*T_RPS2Kp_ BSD* | Present study |
| ***Ogataea polymorpha*** |  | Present study |
| NCYC495 | *leu1.1* |  |

***Supplementary Table 2*.** Relative expression levels of *FMN1*, *rosB*, *rosA* and *rosC* genes in FP/rosB, BRP/FMN1-rosB-rosA-rosC, Y-33/FMN1-rosB and Y-33/FMN1-rosB-rosA-rosC strains versus the recipient strains AF-4, BRP and Y-33. The expression of the *FAD1* gene in *C. famata* and *K. phaffii* was used as a normalization reference for the *rosA, rosB*, and *rosC* genes in the engineered strains*.* The relative expression levels were obtained via the comparative Ct method for quantification of the ΔΔC_t_ values. Error bars indicate standard deviations calculated from at least two independent experiments performed in triplicates. Strains were cultivated on YNB medium supplemented with yeast extract and 2% glucose at 28°C, 220 rpm

| **ΔΔCt** | ***FMN1*** | ***rosB*** | ***rosC*** | ***rosA*** |
| --- | --- | --- | --- | --- |
| FP/rosB / AF-4 | 6.1 ± 0.3 | 20.0 ± 0.6 | NA | NA |
| BRP/FMN1-rosB-rosA-rosC / BRP | 5.3 ± 0.5 | 53.3 ± 1.8 | 46.5 ± 1.7 | 102.3 ± 2.9 |
| Y-33/FMN1-rosB / Y-33 | 6.3 ± 0.1 | 41.3 ± 3.1 | NA | NA |
| Y-33/FMN1-rosB-rosA-rosC / Y-33 | 26.7 ± 1.2 | 161.0 ± 8.3 | 259.0 ±5.4 | 261.0 ± 9.9 |

NA, not applicable

***Supplementary Table 3 List of primers used in this study (restriction sites are underlined)***

| **Primer name** | **Primer sequence 5’-3’** |
| --- | --- |
| IMH3f | Ccg CTC GAG gtc gat ggt ctt gat cat tc |
| IMH3r | CCg CTCGAG GCA TTC TGT TCC AGT TTC TG |
| Ko430 | AAA TCT AGA GAT TAT TGA CTC GAG ATG TTG CGC CG |
| Ko825 | GAT TAC GCC AAG CTT TAA CGA ACA GCT CAT CAG ATT TAC |
| Ko826 | TTA GTC GAC AAG CTT ATA CAT AAT ACC TTA AGT AAA AGT ACC |
| Ko827 | GTA TAA GCT TGT CGA CTA ACG AAC AGC TCA TCA GAT TTA C |
| Ko828 | CCA TGG ATC CTT TGC TTA ATG TAT AAT AAT AGT ATA CTG |
| Ko829 | AAG CAA AGG ATC CAT GGC CTT AAA GGC TCT TAT ATT G |
| Ko830 | GGC CCG TCG ACA TGT TGC GCC GAA CAA TCA CAT |
| Ko831 | CAT GTC GTC GAC GAC GGG CCC GTC GAT GGT CTT GAT CAT TCA |
| Ko832 | GTT AGG ATC CGG TAC CGG GCC CGC ATT CTG TTC CAG TTT CTG TTC |
| Ko833 | GGT ACC GGA TCC TAA CGA ACA GCT CAT CAG ATT TAC |
| Ko834 | GCA TGG ATC CTT TGC TTA ATG TAT AAT AAT AGT ATA CTG |
| Ko835 | AAG CAA AGG ATC CAT GCG TCC TGA GCC AAC AG |
| Ko836 | GCT CGG TAC CAT GTT GCG CCG AAC AAT CAC AT |
| Ko838 | GTT AAA GCT TGG CGT AAT CAT GGT C |
| Ko1035 | TCA AGG CGC GCC ATA TTT AGG GTA TTT TTC TCA C |
| Ko1036 | ATA TGG CGC GCC TTG AAA AGT CAA CTA GTA ATC TT |
| Ko1037 | GCG AAC ACC TTT CCC AAT TTT G |
| Ko1038 | AAT TGA GAT TCT TCA ACC TTA ACT TC |
| Ko1039 | AGC AGA TAT AAA ATG GAG AGC AG |
| Ko1040 | CAT TAT GGA CCT GTT CTT TGT AAC |
| Ko1058 | GTA AAT AGG GTA CAG CCC TAT C |
| Ko1059 | TCT CGG GTG TGT TCT GAT ATG |
| Ko1060 | CAG CAG GTC CTA ACC AAT ATG |
| Ko1061 | CCC ATA GCC TGA CGT ATT CTA A |
| Ko1062 | ACA TGT CGA CGG GCC CAG TCT TAT ATA TAT CCG AAC TTG G |
| Ko1063 | TCC GGT ACC GGG CCC TCA CAT AAC CAC AAG GTG CC |
| Ko1075 | CGC GGA TCC ATG TCT GAT GGT AGA GAA TCT TTT TTA G |
| Ko1076 | GCA CTG CAG TTA AAT AAC ATC AGA TGG AGC AGC AG |
| Ko1077 | CCG GAA TTC AAA TTG ACT GGT CTG AAA TAA TAG |
| Ko1078 | CCG GAA TTC GAT TAT TGA CTC GAG ATG TTG C |
| Ko1079 | CTA AAC CAG TAT AAG AAT CAG CAT G |
| Ko1081 | AAA ACA CCG AAT TCA TGT CTG ATG GTA GAG AAT CTT TTT TG |
| Ko1082 | CTA AAG CGG TAC CTT AAA TAA CAT CAG ATG GAG CAG CAG |
| Ko1083 | ATT TAA GGT ACC GCT TTA GTC AAA TAT TAA TCT ATT TCA CC |
| Ko1084 | GAC ATG AAT TCG GTG TTT TGA TAG TTG TTC AAT TGA TTG |
| Ko1085 | GAA TTC GAG CTC TTT TTT GTA GAA ATG TCT TGG TGT CC |
| Ko1086 | ACT AGG GCC CCT CTA ATC CGG ACT GTC CAA AG |
| Ko1087 | TTA GAG GGG CCC TAG TAA ACA GGG AGA TAC CGT AC |
| Ko1088 | TAC GGT CGA CGT AAG GCA ACT ACA TTA CGA TTG TG |
| Ko1089 | CCT TAC GTC GAC CGT AAG CAA TCT GGA CAA TTA TGT AAG |
| Ko1090 | CCT CTG CAG GTC CAC TAG GCC ACA AGA GTG |
| Ko1091 | GTG GAC CTG CAG AGG CCT GCA TGC AAG CTT G |
| Ko1092 | CAA AAA AGA GCT CGA ATT CAC TGG CCG TCG TTT TAC |
| Ko1093 | CTA ACT CTC AAA CCA TGT TCA GC |
| Ko1140 | TTA GAG CTC GAG CAA TTC GTC TTC TTC AAC AGA GTC |
| Ko1141 | TTA CGC TCG AGG TTC ACA TCA GAA AGG TCA AGC TG |
| Ko1142 | GAT GTG AAC CTC GAG CGT AAG CAA TCT GGA CAA TTA TGT AAG |
| Ko1143 | ACG AAT TGC TCG AGC TCT AAT CCG GAC TGT CCA AAG |
| Ko1145 | GCT CTA GAC GTA AGC AAT CTG GAC AAT TAT GTA AG |
| Ko1146 | ACG CGT CGA CCT CTA ATC CGG ACT GTC CAA AG |
| Ko1147 | ACG CGT CGA CGC TCC CCC CTA CCA AGC CTA C |
| BsaI_FS2 FMN1_Pipa_FW | GAT CGG TCT CAC ATG ACT CGA CCG GTG ATA C |
| BsaI_FS3 FMN1_Pipa_BW | GAT CGG TCT CAA AGC TTA TTT AAC ATT ATG GAC CTG TTC TTT G |
| BsaI_FS2 rosB_syn_FW | GAT CGG TCT CAC ATG GCT TTG AAG GCT TTG ATT TTG |
| BsaI_FS3 rosB_syn_BW | GAT CGG TCT CAA AGC TTA ACC CAA TTG AGA TTC TTC AAC C |
| BsaI_FS2 rosA_syn_FW | GAT CGG TCT CAC ATG CGT CCA GAG CCC ACA GAA C |
| BsaI_FS3 rosA_syn_BW | GAT CGG TCT CAA AGC TCA TCC GGC AGT GCC CCT ACA C |
| RBFa | TAT TGA ACA CAA CCT TGC GTC G |
| RBRa | ATC CTA ACT GAG ATT CCT CAA CC |
| FMN1_qFCf | TTT CCT ATG GTT ATG TCT ATT GG |
| FMN1_qRCf | AAT TCT GGT CTG ATA TAC CCC |
| rosA_qFCf | GAG AAT TAG GTA AGG TCC TTG |
| rosA_qRCf | CAG TTC CTC TAC ATT CTA TAA C |
| rosB_qFCf | ATA TTG GTT AGG ACC TGC TG |
| rosB_qRCf | TGT AAG GTG TCT CTT GAA GC |
| rosC_qF | CTG TTG TTG GTC ATG ATT CTG |
| rosC_qR | GAG TAG CAT CAC CAA TAG ATC |
| ACT1_qFDh | ATG AAG TGT GAT GTC GAT GTC |
| ACT1_qRDh | TTT GAG ATC CAC ATT TGT TGG AA |
| FMN1_qFPp | ATC CAT GTT ATT CAC AAG TTT CC |
| FMN1_qRPp | CTG TTC TTT GTA ACT CTG ATA TC |
| rosA_qFPp | GGT AAA GTG TTG GAT CTA GAC |
| rosA_qRPp | CTC TAT TAC TGC CCA TAC ATC |
| rosB_qFPp | CAT GGT TGC TAA CAC TTC TC |
| rosB_qRPp | TCA ACC TTA ACT TCA CCC AAC |
| ACT1_qFPp | TCT ACA GTA ACA TCG TTA TGT CC |
| ACT1_qRPp | ATT CGT CGT ACT CTT GCT TTG |
| FAD1_qFCf | GAG GAT ATC TGG GAT TTC TTA G |
| FAD1_qRCf | CTC TTT CAT CTG CAT TCT TTC TC |
| FAD1_qFPp | GAA TGG GAT TAC CAC CAA ATT TG |
| FAD1_qRPp | CGG AAT CAT CCA GCA AGT AC |

***Supplementary Sequences***

>rosB optimized for *C. famata*

ATGGCCTTAAAGGCTCTTATATTGAACACAACCTTGCGTCGAAGTCCTTCTCGTTCACAGACACAGGGGTTAATTGACAAGGCCGTACCATTATATGAGAAGGAAGGGATTGAAACTGAAGTTGTCAGAGTGATCGACCATGACATAGAGCAAGAATATTGGGATGACTATGATGATTGGAACGCCGGAGAAAAAGCCCGAAGAGAAGACGAATGGCCATGGCTTTTAGAGAAAATACGAGAAGCTGATATTTTAGTGATAGCTACTCCAATCACTCTTAATATGTGTACCTCCGCTGCCCACGTTATACTTGAAAAATTGAACCTTATGGATGAGCTTAACGGTGACACAAAGCAATTCCCACTTTACAATAAAGTGGCTGGGTTGCTTATGTGCGGTAATGAAGATGGGGCACACCACGTCGCTGGTACCGTTCTTAACAATTTAGGGCGTTTAGGGTATTCCGTCCCACCAAACGCCGCCGCATATTGGTTAGGACCTGCTGGAACCGGGCCAGGATATATTGAGGGAAAAGGGGATCGACACTTCCACACTAACAAATTGATACGTTTTATGGTCGCCAATACTTCACATTTGGCCAGAATGCTTCAAGAGACACCTTACACAACCGATTTAGAAGCATGTGCTCAGGCTGCCAGAGAAGAAAGTGACGACGTGTTTGCCATTCGAGTAAATGTCAATACCCCTGCCATTCGATACAAACGTTTTCAGAAGTTAGGTGAAGTAAAGGTTGAGGAATCTCAGTTAGGATGA

>rosC optimized for *C. famata*

ATGTCTGATGGTAGAGAATCTTTTTTAGAAGTTATGAGATCTGTTTATGAAAGATATTTAGTTGGTGTTCCAGGTGTTTCTGAAGTTTGGTTAATTAGACATGCTGATTCTTATACTGGTTTAGAAGATTATGATGGTGATCCAAGAGATCCAGCTTTATCTGAAAAAGGTAGAGCTCAAGCTAGATTATTAGCTGCTAGATTAGCTGGTGTTCCATTACATGGTGTTTGGGCTTCTGGTGCTCATAGAGCTCAACAAACTGCTTCTGCTGTTGCTGCTGAACATGGTTTAAGAGTTAGAACTGATGCTAGATTAAGAGAAGTTAGAACTAATTGGGATGATGGTAGACCATCTGAATTAAAACCACATGGTGTTTATCCATTTCCAGAACCAGAAAAAGAAGTTGCTGAAAGAATGAGAACTGCTGTTACTGCTGCTGTTGCTGCTACTCCACCAGCTCCAGATGGTACTACTAGAGTTGCTGTTGTTGGTCATGATTCTGCTTTAGTTATTTTAATGGGTTCTTTAATGAATTTAGGTTGGGGTCAATTAGATATGATTTTACCATTAACTTCTGTTTCTGTTTTAGCTGTTAAAGATGAAAGAATGGTTGTTAGATCTATTGGTGATGCTACTCATTTAGCTGCTGCTCCATCTGATGTTATTTAA

>rosA optimized for *C. famata*

ATGCGTCCTGAGCCAACAGAGCATCCTGAAAGAACTGCCGCTCAAAGATTGTACCAGTACAACGTGGATTTGAAGGTCGCCTTCGTGTTATATGCTGTTGCTAAGTTGCACTTACCAGATTTGTTAGCTGACGGGCCTAGAACTACAGCAGACTTGGCTGCCGCCACAGGTAGTGATCCTAGTAGACTTAGAAGACTTTTACGAGCAGCCGCAGGTGCCGATGCTTTGAGAGAGGTCCCAGAAGACTCTTTCGAACTTGCTCCAATGGGTGATTTATTACGTTCTGGTCACCCACGTAGTATGCGTGGAATGACCACCTTCTTCGCAGAGCCTGACGTATTAGCAGCTTACGGGGACTTGGTAGAGAGTGTGCGTACTGGGGTTCCTGCTTTCCAATTACGACACCGAGAACCTTTATACGATTTTTTAGCACGTCCTCAACATAAAGAAGTGCGAGATGAATTTGACGCCGCTATGGTCGAGTTTGGGCAATACTTTGCAGACGATTTCTTAACTTCCTTTGATTTCGGACGATTCACCAGATTCGCTGACATCGGTGGGGGTAGAGGTCAGTTCTTGGCAGGGGTCTTGACCGCCGTTCCATCATCCACAGGTGTTCTTGTTGACGGTCCAGCCGTTGCTGCATCCGCACATAAGTTCCTTGCTTCCCAAAACTTAACCGAAAGAGTAGAAGTCCGAATCGGGGATTTTTTCGATGTGTTACCTACAGGTTGCGACGCCTATGTACTTAGAGGGGTGTTGGAAGATTGGGCAGACGCTGATGCAGTACGACTTTTGGTTAGAATACGTCAGGCTATGGGTGATGCTCCTGAGGCACGATTGTTGATATTAGACTCAGTGATAGGAGAGACCGGAGAATTAGGTAAGGTCCTTGATTTAGACATGTTGGTCTTAGTGGAGGGTGAACATCGTACAAGAGCACAGTGGGACGACCTTCTTGCTCGTGCCGGGTTCGATATTGTAGGGATTCACCCTGCCGGAGATGTTTGGGCCGTTATAGAATGTAGAGGAACTGCTGGTTGA

>rosB optimized for *K.* *phaffii*

ATGGCTTTGAAGGCTTTGATTTTGAACACTACTTTGAGAAGATCCCCATCTAGATCCCAAACTCAAGGTTTGATTGATAAGGCTGTTCCATTGTACGAAAAGGAAGGTATTGAAACTGAAGTTGTTAGAGTTATTGACCATGATATTGAACAAGAATACTGGGATGATTACGATGATTGGAACGCTGGTGAAAAGGCTAGAAGAGAAGATGAATGGCCATGGTTGTTGGAAAAGATTAGAGAAGCTGATATTTTGGTTATCGCTACTCCAATTACTTTGAACATGTGTACTTCTGCTGCTCATGTTATTTTGGAAAAGTTGAACTTGATGGATGAATTGAACGGTGATACTAAGCAATTTCCATTGTACAACAAGGTTGCTGGTTTGTTGATGTGTGGTAACGAAGATGGTGCTCATCATGTTGCTGGTACTGTTTTGAACAACTTGGGTAGATTGGGTTACTCTGTTCCACCAAACGCTGCTGCTTACTGGTTGGGTCCAGCTGGTACTGGTCCAGGTTACATTGAAGGTAAGGGTGATAGACATTTTCATACTAACAAGTTGATCAGATTCATGGTTGCTAACACTTCTCATTTGGCTAGAATGTTGCAAGAAACTCCATACACTACTGATTTGGAAGCTTGTGCTCAAGCTGCTAGAGAAGAATCTGATGATGTTTTTGCTATTAGAGTTAACGTTAACACTCCAGCTATTAGATACAAGAGATTTCAAAAGTTGGGTGAAGTTAAGGTTGAAGAATCTCAATTGGGTTAA

>rosC optimized for *K.* *phaffii*

ATGTCTGATGGTAGAGAATCTTTTTTGGAAGTTATGAGATCTGTTTACGAAAGATACTTGGTTGGTGTTCCAGGTGTTTCTGAAGTTTGGTTGATTAGACATGCTGATTCTTACACTGGTTTGGAAGATTACGATGGTGATCCAAGAGATCCAGCTTTGTCTGAAAAGGGTAGAGCCCAAGCTAGATTGTTGGCTGCTAGATTGGCTGGTGTTCCATTGCATGGTGTTTGGGCTTCTGGTGCTCATAGAGCCCAACAAACTGCTTCTGCTGTTGCTGCTGAACATGGTTTGAGAGTTAGAACTGATGCTAGATTGAGAGAAGTTAGAACTAACTGGGATGATGGTAGACCATCTGAATTGAAGCCACATGGTGTTTACCCATTTCCAGAACCAGAAAAGGAAGTTGCTGAAAGAATGAGAACTGCTGTTACTGCTGCTGTTGCTGCTACTCCACCAGCTCCAGATGGTACTACTAGAGTTGCTGTTGTTGGTCATGATTCTGCTTTGGTTATTTTGATGGGTTCTTTGATGAACTTGGGTTGGGGTCAATTGGATATGATTTTGCCATTGACTTCTGTTTCTGTTTTGGCTGTTAAGGATGAAAGAATGGTTGTTAGATCTATTGGTGATGCTACTCATTTGGCTGCTGCTCCATCTGATGTTATTTAA

>rosA optimized for *K.* *phaffii*

ATGCGTCCAGAGCCCACAGAACACCCCGAAAGAACAGCTGCCCAGAGGTTGTACCAATATAACGTGGATTTGAAAGTCGCCTTTGTGTTGTATGCAGTGGCAAAACTTCACCTACCCGATCTACTGGCCGACGGTCCAAGGACGACGGCTGACTTGGCAGCCGCAACCGGCTCTGACCCTTCTAGATTAAGGAGATTACTAAGAGCCGCCGCAGGTGCCGACGCCTTAAGAGAGGTACCCGAAGATTCTTTTGAACTAGCTCCAATGGGAGATCTGTTAAGATCTGGCCATCCTCGTTCTATGCGTGGTATGACTACATTCTTCGCAGAGCCAGATGTCCTGGCTGCCTACGGCGATTTAGTTGAGTCCGTTAGGACTGGCGTTCCCGCATTTCAATTAAGGCACAGGGAACCTTTGTATGATTTTTTGGCCAGACCTCAACACAAGGAGGTGCGTGATGAATTCGATGCTGCTATGGTTGAGTTCGGTCAATACTTCGCAGACGACTTTCTAACATCATTCGATTTCGGCAGGTTCACAAGGTTTGCAGACATTGGCGGTGGAAGGGGCCAGTTTCTAGCTGGCGTCTTGACTGCTGTCCCTAGTAGTACTGGCGTTCTGGTCGACGGTCCAGCCGTCGCTGCTTCAGCACATAAATTCCTGGCCTCACAGAACCTGACAGAGAGAGTTGAGGTGAGGATCGGCGATTTTTTCGATGTATTGCCCACAGGCTGCGATGCATATGTTTTGCGTGGTGTTTTAGAGGATTGGGCTGACGCTGACGCTGTTCGTTTACTGGTGAGGATTAGACAAGCTATGGGTGACGCACCCGAGGCTAGACTGCTGATCCTAGACTCAGTCATCGGCGAGACGGGAGAGCTAGGTAAAGTGTTGGATCTAGACATGCTAGTACTAGTTGAAGGTGAGCACCGTACCAGGGCACAATGGGACGATCTGCTGGCTAGAGCTGGTTTTGACATCGTTGGCATACATCCTGCCGGAGATGTATGGGCAGTAATAGAGTGTAGGGGCACTGCCGGATGA
